# Genome-wide association mapping in a wild avian population identifies a link between genetic and phenotypic variation in a life-history trait

**DOI:** 10.1098/rspb.2015.0156

**Published:** 2015-05-07

**Authors:** Arild Husby, Takeshi Kawakami, Lars Rönnegård, Linnéa Smeds, Hans Ellegren, Anna Qvarnström

**Affiliations:** 1 Department of Animal Ecology, Evolutionary Biology Centre (EBC), Uppsala University, Norbyvägen 18D, Uppsala 75236, Sweden; 2 Department of Evolutionary Biology, Evolutionary Biology Centre (EBC), Uppsala University, Norbyvägen 18D, Uppsala 75236, Sweden; 3 Centre for Biodiversity Dynamics, Department of Biology, Norwegian University of Science and Technology, Trondheim 7491, Norway; 4 Department of Biosciences, University of Helsinki, PO Box 65, Helsinki 00014, Finland; 5 Department of Clinical Sciences, Swedish University of Agricultural Sciences, Uppsala 75007, Sweden

**Keywords:** clutch size, egg production, *Ficedula albicollis*, fitness trait, GWAS, QTL

## Abstract

Understanding the genetic basis of traits involved in adaptation is a major challenge in evolutionary biology but remains poorly understood. Here, we use genome-wide association mapping using a custom 50 k single nucleotide polymorphism (SNP) array in a natural population of collared flycatchers to examine the genetic basis of clutch size, an important life-history trait in many animal species. We found evidence for an association on chromosome 18 where one SNP significant at the genome-wide level explained 3.9% of the phenotypic variance. We also detected two suggestive quantitative trait loci (QTLs) on chromosomes 9 and 26. Fitness differences among genotypes were generally weak and not significant, although there was some indication of a sex-by-genotype interaction for lifetime reproductive success at the suggestive QTL on chromosome 26. This implies that sexual antagonism may play a role in maintaining genetic variation at this QTL. Our findings provide candidate regions for a classic avian life-history trait that will be useful for future studies examining the molecular and cellular function of, as well as evolutionary mechanisms operating at, these loci.

## 1. Introduction

Life-history traits such as timing of maturation, fecundity and survival are important components of the long-term fitness of individuals [1,2]. A classic life-history trait closely associated with fitness in many species of animals is clutch size, the number of eggs produced by a female during a reproductive event [3–5]. The evolution of optimal clutch size has been extensively studied in the light of life-history theory, especially in relation to the central trade-offs between number and quality of offspring, and between current and future reproduction [3,6–8]. For example, some studies have found that experimentally increased clutch size leads to decreased quality of each individual young [9] or reduced adult survival [10].

A long-standing paradox that needs resolution is the observation that life-history traits, such as clutch size, often seem to harbour abundant genetic variation despite being under directional or stabilizing selection [11]. There are a number of theoretical models showing how genetic variation in fitness traits can be maintained at the population level [12], but empirical tests are needed before any general conclusions can be made. This is especially true for assumptions about selective forces acting on the causal genetic variants underlying fitness traits, because processes such as over-dominance and intra-locus conflict can contribute to the maintenance of genetic variation, but are easily overlooked when focusing on the phenotypic level. Detecting fitness loci and the patterns of selection acting on them is therefore an important goal in evolutionary genetics as it contributes to a more detailed understanding of the mechanisms of evolution [13–17].

While previous studies on birds have demonstrated significant heritability of clutch size (e.g. [4,18,19]), the genes and the molecular and cellular processes generating inter-individual variation in clutch size are not yet known. A first step towards a more mechanistic understanding of how clutch size is regulated in natural populations is to identify genomic regions that harbour genetic variants for clutch size. Conducting genome-wide scans has become greatly facilitated by recent advances in sequencing and genotyping technology [20,21], which have significantly broadened the range of organisms amenable to genetic mapping studies [15,22,23]. Here, we take advantage of such developments to carry out genome-wide association mapping in a wild population of collared flycatchers (*Ficedula albicollis*), a small migratory passerine that breeds in eastern and central Europe, and is an important ecological model organism [24,25]. Specifically, we benefit from the availability of the genome sequence for the collared flycatcher [26] and a custom 50 k SNP array spanning most of the genome of this species [27,28] to map genomic regions governing variation in clutch size.

## 2. Material and methods

### (a) Study population and collection of data on reproductive performance

Individuals included in this study were breeding on the island of Öland during the years 2003–2010 and were monitored as part of a long-term study on pied (*F. hypoleuca*) and collared flycatchers [25]. A range of phenotypic and life-history characters are measured yearly according to a standard field protocol, and a blood sample is taken from all breeding individuals and their offspring for subsequent genetic analyses.

### (b) Genotyping

Eight hundred and sixty-four collared flycatchers were genotyped with a custom-made 50 k Ilumina iSelect BeadChip with 45 138 SNPs successfully included on the chip [27]. For further information about array construction and performance, see [27]. Out of the 864 individuals genotyped, data on clutch size and lifetime reproductive success (LRS) from 313 females were available for genome-wide association study (GWAS; see below).

### (c) Genome-wide association analysis

After removing markers with a call rate of less than 95%, minor allele frequency (MAF) of less than 0.01 and a *p*-value for rejection of Hardy–Weinberg equilibrium (HWE) of less than 0.001, we had 37 309 SNPs available for downstream analysis. Markers deviating from expected HWE were removed to safeguard against potential genotyping errors [29]. Ten individuals were removed prior to analyses due to disagreement between observed (phenotypic) and molecularly defined sex.

Current statistical methods for GWAS do not allow including repeated measures on the same individual [30], and we therefore developed a novel statistical method for fitting both repeated measures and the relatedness between individuals in the same GWAS model (see the electronic supplementary material). Briefly, we fitted the following linear mixed effect model:
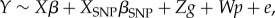
where *X* is the fixed-effect design matrix for non-genetic fixed effects (age and year) and *β* is the corresponding fixed effects, *X*_SNP_ is the SNP-covariate (coded 0, 1, 2) and *β*_SNP_ is the SNP fixed effect. The model includes a random genetic effect *g* and a permanent environmental effect *p* on each individual that are linked to observations by incidence matrices *Z* and *W*, respectively. For further details regarding the fitted model, see the electronic supplementary material. The estimated kinship matrix is the proportion of shared alleles identical by state across all markers weighted by the allele frequencies [31]. Reported *p*-values are from the above model based on Wald tests, and are corrected for relatedness among individuals and the repeated observations on the same individual in the sample. Genome-wide significance threshold was estimated by dividing the significance value (chosen here as 0.05) by the number of markers (37 309), resulting in a significance threshold of *p* = 1.34 × 10^−6^, which is conservative because it assumes all markers are independent. Similarly, a suggestive threshold was estimated allowing for one false positive, resulting in a threshold of *p* = 2.68 × 10^−5^.

The additive effect of a marker was calculated as *V*_SNP_ = 2*pq*(*a* + *d*(*q* − *p*))^2^, where *a* is half the difference between the two homozygotes (genotypic value), *d* is the dominance deviation (which in our case is zero because an additive model was fitted), *p* is the MAF and *q* the major allele frequency [32].

Linkage disequilibrium (LD; measured as *r*^2^) was calculated between markers within the candidate regions including all genotyped individuals using PLINK [33].

### (d) Fitness analyses at QTLs

We examined the association between the genotype at the three candidate loci and LRS in males and females using a generalized linear model with Poisson error structure using R. Lifetime reproductive success was fitted as a response variable and the genotype at the candidate locus was fitted as a three-level factor with inferences about differences in fitness between genotypes evaluated using an ANOVA table. Information on LRS from genotyped individuals was available from the same 313 females as for the analysis of clutch size above and from 301 males that were also genotyped on the SNP array. Similarly, associations between genotype and annual reproductive success was examined using a generalized linear mixed model (GLMM) with Poisson error structure fitting individual identity as a random effect to account for repeated observations across years on the same individuals. For these analyses, we had information from 815 records from 301 males and 656 records from 313 females.

## 3. Results

We first estimated the narrow-sense heritability of clutch size using the realized genomic kinship between individuals in an ‘animal model’ to partition the phenotypic variance in clutch size, which gave an estimate of *h*^2^ = 0.14 (±0.03) (see table 1).

**Table 1.** Quantitative genetic estimates of the variance components from the repeated-measures GWAS model along with their 95% confidence interval (CI) estimated using the delta method [34].

variance component	estimate	CI
*V*_A_ (additive genetic)	0.113	0.079–0.162
*V*_PE_ (permanent environment)	0.086	0.058–0.127
*V*_R_ (residual variance)	0.594	0.524–0.673

**Figure 1.**
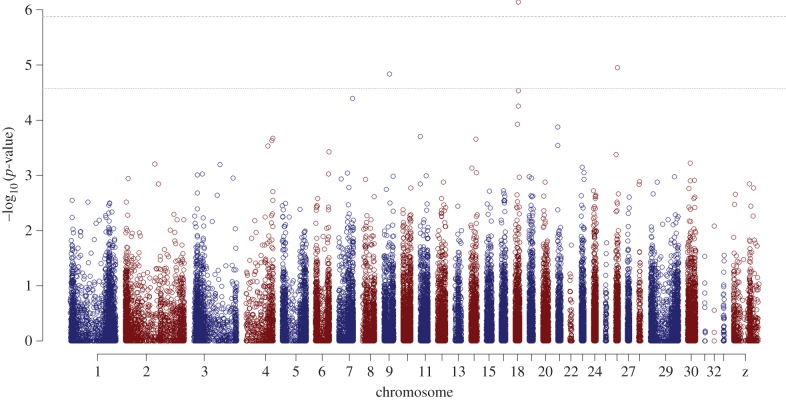
Manhattan plot with –log_10_ *p*-values from the repeated measures screen for the association between marker genotype and clutch size for all 37 309 SNPs. The dashed line indicates the genome-wide Bonferroni-corrected significant threshold and the dotted line the threshold for suggestive significant associations. (Online version in colour.)

**Figure 2.**
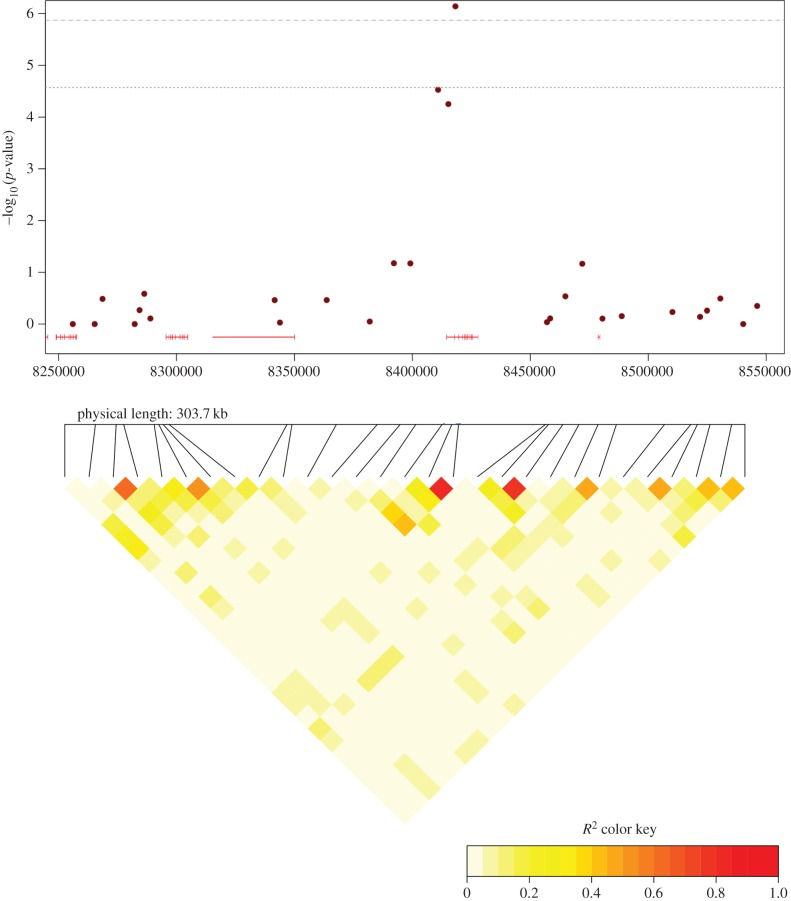
A local Manhattan plot for the approximate 300 kb region on chromosome 18 for which a genome-wide significant QTL for clutch size was located and a heat map representation of the pairwise LD between markers within this region. Dashed line indicates the genome-wide Bonferroni-corrected significant threshold and the dotted line the threshold for suggestive significant associations. Red lines indicate intron–exon boundaries. (Online version in colour.)

Having established a genetic basis to clutch size, we next used a novel repeated-measures GWAS method (see the electronic supplementary material) to detect loci where marker genotype was significantly correlated with observed clutch size, while controlling both for the realized relatedness between individuals as well as the repeated records on individuals (genomic inflation factor *λ* = 1.008; see electronic supplementary material, figure S1). After adjustment for multiple testing using strict Bonferroni correction, we found one SNP on chromosome 18 that was genome-wide significant (marker *N00072* : *1137698*, *p* = 7.23 × 10^−7^; table 2 and figure 1). Moreover, two markers nearby (3 kb and 4.3 kb) also showed a strong, but non-significant, association with clutch size (table 2) and were in relatively strong LD with the significant marker (figure 2). Together, these three markers are located in a small (7 kb) region on chromosome 18 (figure 2) where the closest of our markers upstream from this region is 11.7 kb away and the closest marker downstream is 38.8 kb away. Neither of the markers on each side of the 7 kb QTL region showed an indication of being associated with clutch size (figure 2). As a result, there is a genomic region of 60 kb in which is located the SNP significant at genome-wide level that is of further interest. In addition to the QTL located on chromosome 18, one marker on chromosome 9 (*N00007* *:* *7983448*; table 2) and one on chromosome 26 (*N00075* *:* *2292331*; table 2) were significant at the suggestive genome-wide threshold (see figure 1). For the QTL on chromosome 9, the nearest upstream marker was 56.8 kb away and the nearest downstream marker was 1.6 kb away, and neither of these markers showed any sign of being associated with clutch size. Similarly, the intermarker distance for the QTL on chromosome 26 spanned a 50 kb interval with the nearest marker upstream 17.7 kb away and the nearest marker downstream 25.8 kb away. Again, neither of these two closest markers showed any sign of being associated with clutch size.

**Table 2.** The top 10 markers most highly associated with clutch size in the repeated-measures GWAS. SNP name, chromosome number, chromosomal position, reference allele, coded allele and allele frequency for the reference allele along with estimated effect size, standard error, *p*-value corrected for genomic inflation and call rate. The genome-wide significant marker is highlighted in bold and the suggestive markers in italics.

SNP name	chromosome number	chromosome position	major allele	minor allele	minor allele frequency	effect size	s.e.	*p*-value	call rate
***Chr18_N00072:1137698***	**18**	**8431907**	**A**	**G**	**0.03**	**−0.819**	**0.167**	**7.23 × 10^−7^**	**1.00**
*Chr26_N00075* *:* *2292331*	*26*	*4868665*	*A*	*G*	*0.41*	−*0.277*	*0.063*	*1.13* × *10^−5^*	*1.00*
*Chr9_N00007* *:* *7983448*	*9*	*15102041*	*A*	*G*	*0.32*	*0.289*	*0.068*	*1.47* × *10^−5^*	*1.00*
*Chr18_N00072* *:* *1130347*	18	8424558	G	A	0.08	−0.458	0.109	2.96 × 10^−5^	1.00
*Chr7_N00016* *:* *11337254*	7	36319720	G	A	0.24	−0.285	0.069	4.08 × 10^−5^	1.00
*Chr18_N00072* *:* *1134699*	18	8428909	G	A	0.04	−0.630	0.155	5.60 × 10^−5^	1.00
*Chr18_N00068* *:* *3727934*	18	6183407	G	A	0.08	−0.389	0.100	1.18 × 10^−4^	1.00
*Chr21_N00232* *:* *274271*	21	467752	G	A	0.12	0.399	0.104	1.34 × 10^−4^	1.00
*Chr11_N00156* *:* *109507*	11	90045	A	G	0.07	−0.426	0.114	1.99 × 10^−4^	1.00
*Chr4_N00190* *:* *864394*	4	71299723	A	G	0.21	−0.267	0.072	2.11 × 10^−4^	1.00

These results are robust to the choice of method because a ‘standard’ GWAS approach using the mean clutch size of a female as phenotype (i.e. no repeated measures on the same individual) identified the same QTL region on chromosome 18 and the two suggestive QTLs as the most significant association signals (electronic supplementary material, table S1).

**Figure 3.**
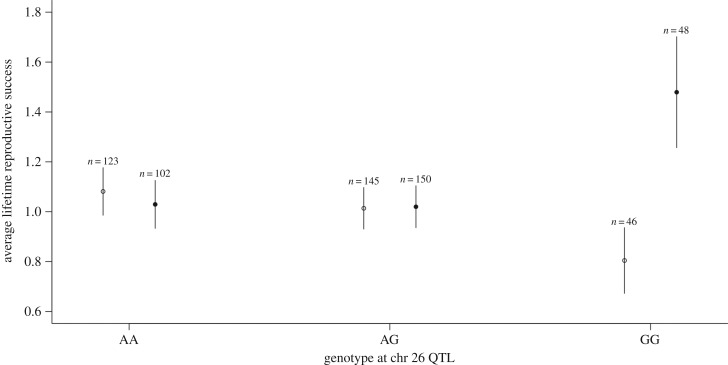
Means and standard errors for LRS for each genotype class in males (filled circles) and females (open circles) at the suggestive QTL on chromosome 26. A significant sex-by-genotype interaction suggests that sexual antagonism may play a role in maintaining genetic variation at this locus.

To learn more about the evolutionary processes operating at the detected QTLs, we examined selection acting among the genotypes in both sexes using LRS as a composite estimate of fitness. As we study a wild population, we can investigate the selection pressures operating on these QTLs within the natural habitat experienced by the birds. For the QTLs at chromosomes 18 and 9, there were no differences in LRS between genotypes in males (chr 18: 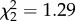, *p* = 0.53; chr 9: 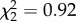, *p* = 0.63) or females (chr 18: 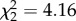, *p* = 0.13; chr 9: 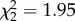, *p* = 0.38). Interestingly, however, for the QTL at chromosome 26, males homozygous for the G allele had significantly higher LRS compared with the other genotypes (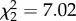, *p* = 0.03; figure 3), whereas this relationship was reversed in females, where individuals homozygous for the same allele had lower LRS, thereby generating a significant interaction between genotype and sex (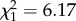, *p* = 0.01; figure 3). The low LRS of GG females seems to be mainly driven by a reduced clutch size (negative effect of the allelic substitution; table 2), which in turn leads to fewer annual fledglings (GLMM: *b* = −0.37, s.e. = 0.16, *t* = 2.32, *p* = 0.02) and therefore reduced LRS (figure 3). In males, there was no difference in annual number of recruited offspring (0.44 recruits compared with 0.37 for the other two genotypes; *p* = 0.13) or lifespan (1.34 calendar years for GG males compared with 1.13 for AA and 1.07 for heterozygotes; *p* = 0.50).

## 4. Discussion

Clutch size represents a classic avian life-history trait and is expected to have a complex genetic basis. We used an ‘animal model’ [35] with the realized genomic kinship between individuals calculated from the genotype data to estimate a narrow-sense heritability of clutch size at *h*^2^ = 0.14 (table 1). This estimate is lower compared with a previous study on the genetics of clutch size in this species in a different population [18], although it is difficult to say if this is due to population differences in genetic architecture or methodological aspects (genomic relatedness used here versus expected relatedness from a social pedigree used in the earlier study).

A general expectation for traits closely related to fitness, such as clutch size, is that genetic variance should be low compared with traits that are less closely associated with fitness [36]. How quickly genetic variance is eroded will depend (among other things) on the number and effect size of the loci underlying the trait, with loci with the largest phenotypic effect becoming fixed first [37]. One prediction is therefore that genetic variation in life-history traits should be governed largely by many loci of small effect [38]. However, population genetic models have shown that in finite populations, this prediction depends on both patterns of recombination and the strength of selection under a migration–selection balance model [39]. For instance, in the absence of migration, a negative exponential distribution of effect sizes of loci underlying fitness traits has been demonstrated [37]. While such a pattern has been observed in studies on *Drosophila* [40], QTL mapping studies on natural populations have typically found few loci with large effect and only rarely have small-effect loci been detected [41]. While this may well be related to the reduced power to detect loci of small effect in studies on natural populations, a genetic architecture with few large-effect QTLs is also predicted by theoretical models for traits under migration–selection–drift balance [39] and for traits that are under weak or strong stabilizing selection [42]. Before any general conclusions can be made regarding these different predictions regarding effect size distributions in natural populations, we need more studies using higher marker density and sample sizes than have been used in the past. In this study, the single genome-wide significant marker detected on chromosome 18 contributed 3.9% of the phenotypic variance, and the suggestive markers on chromosome 26 and 9 contributed 3.6% and 3.7% of *V*_P_, respectively, and as such could be considered loci of large effect. However, the limited sample size in our study (*n* = 313) means effects are clearly overestimated [41,43] and the actual effect size is substantially smaller, although it is not possible to determine the degree of overestimation. This also means it is difficult to draw conclusions about the number of loci underlying clutch size, although we consider it most likely that clutch size in the collared flycatcher has a polygenic basis, similar to that seen for many quantitative traits in model organisms [40]. A polygenic basis would also be in agreement with a recent study that examined the genetic basis of clutch size in great tits (*Parus major*), and found a strong positive correlation between chromosome size and the proportion of genetic variance in clutch size attributed to that chromosome [44]. In that study, no genome-wide significant loci were detected for clutch size, suggesting that many loci with small effect contribute to clutch size.

We detected one genome-wide significant and two suggestive QTLs that were associated with variation in clutch size, and examined whether the detected QTL regions contained any potential candidate genes. The QTL region on chromosome 18 overlaps with the gene *RAB11FIP4* (RAB11 Family Interacting Protein 4), which is needed for the completion of cytokinesis [45–47]. *FIP4* is part of the class II FIPs (*FIP4* and *FIP3*) that localize to the cleavage furrow/midbody during cytokinesis and are probably required for abscission, the final separation of the two cells [48]. The suggestive region found on chromosome 26 was intergenic and any putative biological function is unknown. The other suggestive region on chromosome 9 was within the intron of the *Urotensin* (*UTS2B/D*) gene, which is important for vasoconstriction in regulation of blood pressure [49]. Future functional work will be needed to resolve the potential role of these genes in contributing to clutch size variation.

In general, there were few fitness differences among genotypes at the identified QTLs when examining a more complete measure of fitness than clutch size, LRS. While we did discover an interesting sex-by-genotype interaction for LRS at the QTL on chromosome 26, it is important to keep in mind this is a suggestive QTL. Nevertheless, the preliminary analyses indicate that intra-locus conflict may play some role in maintaining genetic variation at this locus because females homozygous for the G allele at this QTL had significantly lower LRS compared with GG males (figure 3). That the same genotype at this locus has sex-specific effect on LRS seems to be a combined result of slightly higher annual reproductive success and longer lifespan in males, and lower clutch size and fledgling production in females. However, future studies will need to address this question in more detail and its possible role in maintaining genetic variation at this locus.

Previous studies that have detected fitness differences among genotypes at adaptive loci, such as horn morphology in Soay sheep [50] or armour plating in three-spined sticklebacks [51], have been on traits where the genetic basis is Mendelian or near Mendelian. For such traits, effect size differences between genotypes are expected to be larger, and therefore easier to detect, than for loci underlying a quantitative trait, as studied here.

QTL studies on complex traits in natural populations are rare [52–58] and, as a result, we know comparatively little about the genetic underpinnings of some of the most commonly observed traits in nature. Our study identified three genomic regions of interest associated with clutch size and indicated opposing fitness effects between the sexes for the QTL at chromosome 26, suggesting that sexual antagonism may contribute to the maintenance of genetic variation at this locus. This opens the possibility for future studies to examine mechanisms that can maintain genetic variation at the locus level and to examine the functional role of the genes discovered in these QTL regions.

## Supplementary Material

Supplementary material

## Data accessibility

All data used are available from Dryad (http://dx.doi.org/10.5061/dryad.sm1vt).
