# Supplementary material for: Genome-wide association mapping in a wild avian population identifies a link between genetic and phenotypic variation in a life-history trait
Source: Proc Biol Sci. 2015 May 7;282(1806):20150156. doi: 10.1098/rspb.2015.0156 (PMC4426624; doi:10.1098/rspb.2015.0156)
Supplement: Supplementary material [file rspb20150156supp1.docx]

SUPPLEMENTARY MATERIAL

Genome-wide association mapping in a wild avian population identifies a link between genetic and phenotypic variation in a life history trait

Arild Husby^1,2,5*^, Takeshi Kawakami^3^, Lars Rönnegård^4^, Linnéa Smeds^3^, Hans Ellegren^3^ and Anna Qvarnström^1^.

^1^ Department of Animal Ecology, Evolutionary Biology Centre (EBC), Uppsala University, Norbyvägen 18D, SE-75236 Uppsala, Sweden

^2^ Centre for Biodiversity Dynamics, Department of Biology, Norwegian University of Science and Technology, N-7491 Trondheim, Norway.

^3^ Department of Evolutionary Biology, Evolutionary Biology Centre (EBC), Uppsala University, Norbyvägen 18D, SE-75236 Uppsala, Sweden.

^4^ Department of Clinical Sciences, Swedish University of Agricultural Sciences, SE-75007 Uppsala, Sweden.

^5^ Department of Biosciences, University of Helsinki, P.O. Box 65, FI-00014 Helsinki, Finland

* Author for correspondence: Arild Husby: arild.husby@helsinki.fi

Figure S1: Q-Q plot of observed versus expected χ^2^ value for the repeated measures GWAS. There was no indication of genomic inflation (λ= 1.008) after the genomic kinship matrix was fitted in the model.


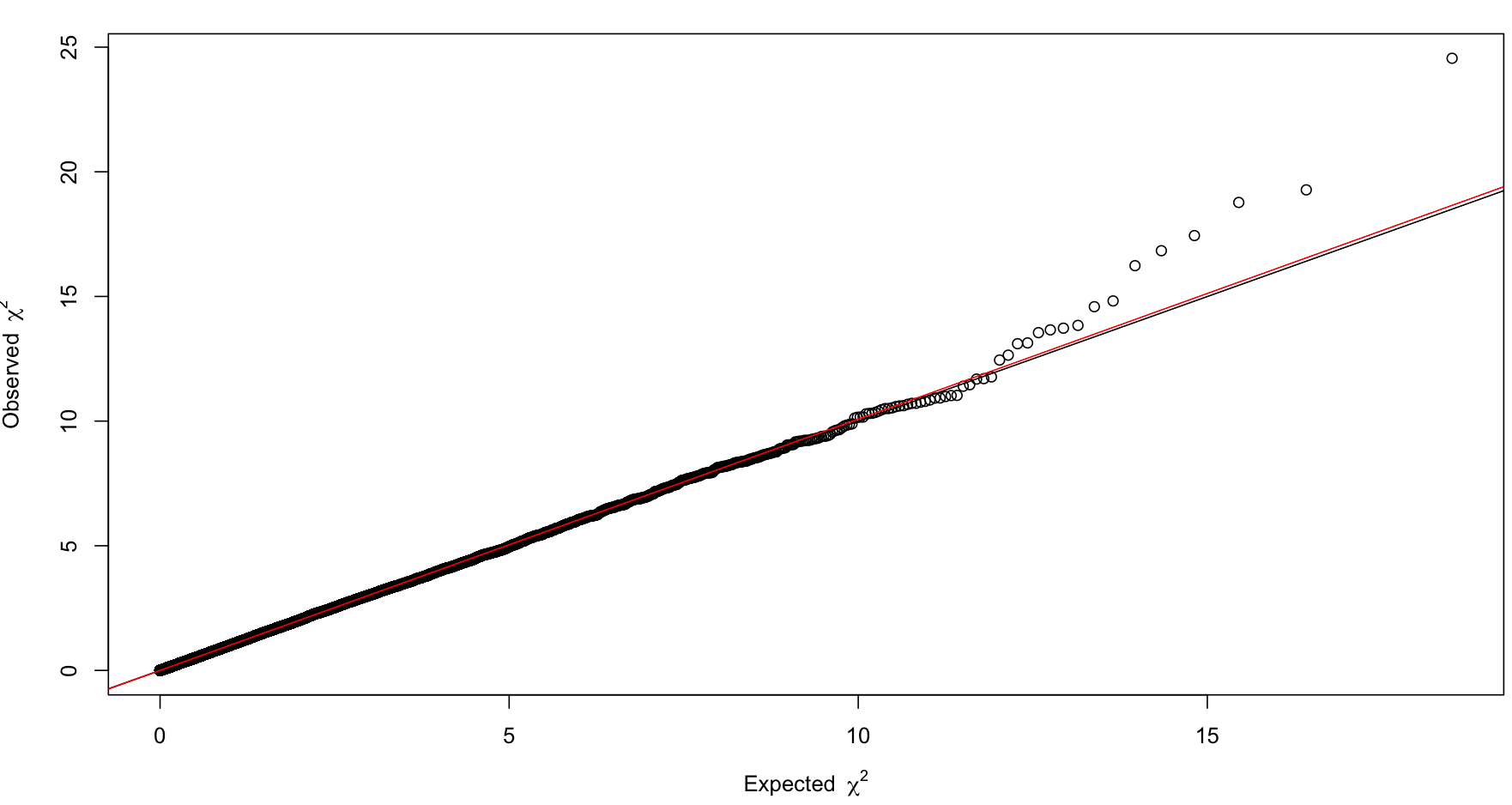


**Table S1:** The top ten markers most highly associated with mean clutch size in the GWAS. SNP name, chromosome number, chromosomal position, the reference allele, coded allele and allele frequency for the reference allele along with estimated effect size, standard error, P-value corrected for genomic inflation and call rate.

| **SNP name** | **Chromosome number** | **Chromosome position** | **Major allele** | **Minor allele** | **Minor allele frequency** | **Effect size** | **Standard error** | **P-value** | **Call rate** |
| --- | --- | --- | --- | --- | --- | --- | --- | --- | --- |
| *Chr18_N00072:1130347* | 18 | 8424558 | G | A | 0.08 | -0.56 | 0.11 | 1.22E-06 | 1.00 |
| *Chr18_N00072:1137698* | 18 | 8431907 | A | G | 0.03 | -0.82 | 0.17 | 2.40E-06 | 1.00 |
| *Chr26_N00075:2292331* | 26 | 4868665 | A | G | 0.41 | -0.28 | 0.06 | 2.17E-05 | 1.00 |
| *Chr18_N00072:1134699* | 18 | 8428909 | G | A | 0.04 | -0.65 | 0.16 | 6.53E-05 | 1.00 |
| *Chr9_N00007:7983448* | 9 | 15102041 | A | G | 0.32 | 0.29 | 0.07 | 7.43E-05 | 1.00 |
| *Chr7_N00016:11337254* | 7 | 36319720 | G | A | 0.24 | -0.28 | 0.07 | 0.000129247 | 1.00 |
| *Chr12_N00057:1784423* | 12 | 9278801 | A | C | 0.20 | -0.32 | 0.08 | 0.000130601 | 1.00 |
| *Chr23_N00040:7193858* | 23 | 7702055 | A | G | 0.41 | 0.24 | 0.06 | 0.000214648 | 1.00 |
| *Chr5_N00062:4758164* | 5 | 18115819 | A | G | 0.08 | -0.42 | 0.11 | 0.000233795 | 1.00 |
| *Chr18_N00068:2447455* | 18 | 4903090 | G | A | 0.05 | 0.57 | 0.15 | 0.000243042 | 1.00 |
